# Genomica: linear mixed model based, multiple hypothesis testing corrected, ortholog functional enrichment analysis

**DOI:** 10.1186/s12859-026-06450-y

**Published:** 2026-04-18

**Authors:** Salvatore Galgano

**Affiliations:** Monogastric Science Research Centre, School of Veterinary Medicine and Biosciences, Scotland’s Rural College (SRUC), Edinburgh, UK

**Keywords:** Bioinformatics, Differential, Abundance Analysis, Metagenomics, Microbiota, Ortholog, KEGG

## Abstract

**Background:**

The analysis of ortholog genes derived from metagenomic experiments provides an invaluable opportunity to assess the functional role of microbial communities towards, for example, antimicrobial resistance or biochemical pathways under different experimental conditions. Nevertheless, the integration of the statistical analysis of these complex data sets and the enrichment of the derived significantly differential abundant orthologs is not currently facilitated by existing software. Genomica is an R package that, with minimal input from the user, allows to perform a double-step analysis of functional orthologs from the KEGG Orthology. The pipeline is carried out via combining false discovery rate corrected linear mixed models to functional enrichment analysis through integrating established R pipelines (i.e., lme4 and MicrobiomeProfiler).

**Results:**

Only two data frames are needed as input to run Genomica, which contain data and metadata, respectively. The fast pipeline integrated within the function Genomica allows to analyze 4000 orthologs in circa 3 min. The outputs are collected in a single directory, containing publication-ready results from the linear mixed model and from the enrichment analysis. The Benjamini & Hochberg correction is applied to the results from the linear mixed model, therefore only P adjusted significant comparisons are further included in the enrichment analysis.

**Conclusions:**

Genomica is a simple-to-use R package to analyze complex datasets, integrating a well-founded statistical analysis, accounting for the calculation of the type I error under repeated testing, with the enrichment analysis of the significantly differential abundant orthologs across experimental conditions, all with minimal input from the user.

**Supplementary Information:**

The online version contains supplementary material available at 10.1186/s12859-026-06450-y.

## Background

The advancement of metabarcoding and metagenomic has paved the way to the analysis of unculturable microbial communities and their genomes with an unprecedented level of detail [1]. Both short-read and long-read sequencing technologies have been vastly implemented throughout the last decade [2]. This allowed the in-depth analysis of the microbiota (i.e., microorganisms present in a determined environment), the metagenome (i.e., microbial genomes) and of the entire habitat comprising microbiota, metagenome and environmental conditions (i.e., microbiome) [3]. Consequently, several bioinformatic pipelines have been developed to analyze metagenomic sequences [4], implementing both direct profiling reference-based protocols and de novo assembly [5]. Independently of the bioinformatic approach used, metagenomic functional characterization enables to unravel the microbial potential towards specific functions or pathways, and grouping genes into orthologs is a well-established way to explore these functions [6]. Orthologs are prokaryotic or eukaryotic genes descending from a common ancestor by a speciation event [7], whose analysis in metagenomic settings can reveal pivotal information about processes, for example related to antimicrobial resistance [8] or biochemical functions such as carbohydrate degradation [9]. A substantial contribution to the study of the orthologs has been provided by the establishment of the Kyoto Encyclopaedia of Genes and Genomes (KEGG), with the aim of cataloguing functional annotations at gene level, in which KEGG orthologs (KOs) are stored according to their existence within defined functional groups [10].

Functional enrichment analysis (FEA) allows to identify over-represented biological functions within a gene list, by statistically comparing the latter to a background gene population [11]. One of the packages specifically designed for metagenome functional enrichment analysis is MicrobiomeProfiler [12, 13]. MicrobiomeProfiler is a remarkable tool, and the authors suggest a number of protocols to statistically pre-analyze data in order to classify differentially abundant genes within complex datasets before the enrichment [14], such as the implementation of DESeq2 for transcriptomic data [15]. Nevertheless, the suggested methods require to carry out the statistical analysis as a further step, separately from the enrichment. This might lead to de-standardize protocols as these are carried out by different groups. On the other hand, there are only a few examples of software that carry out statistical analysis of these complex data sets, such as MaAsLin2 [16], ANCOM-BC [17] and DESeq2, which however do not include FEA within their pipeline.

Genomica is a single-function, easy-to-use R package which integrates statistical and enrichment analysis. The only required input files are a gene or feature table (e.g., pre-normalized KO abundance across different samples) and a metadata table. The outputs of Genomica are summarized in publication-ready tables and figures. This is possible, thanks to the integration of multiple testing via linear mixed models (LMM) and the FEA through implementing lme4 [18] and MicrobiomeProfiler, respectively.

The current version of Genomica is 2.0.1. It allows users to specify the significance threshold below which adjusted *p* values are considered significant and to manually define the resolution of the output figures. Furthermore, Genomica v2.0.1 performs LMM diagnostics by testing for residual normality, heteroscedasticity and outliers. These diagnostic procedures enable users to independently evaluate the adequacy of the statistical model and determine whether the applied analytical approach is appropriate for their specific datasets.

### Implementation

**Fig. 1:**
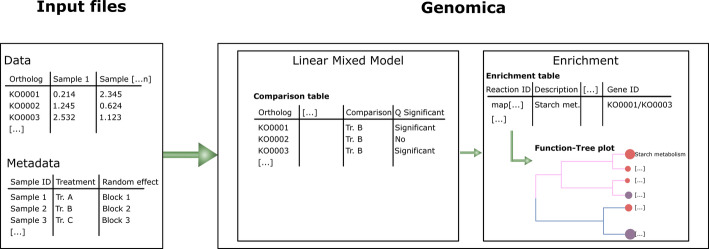
Example of pipeline execution in Genomica. The required input files are a Data table (e.g., KO normalized abundance across the samples) and a Metadata table, in which users can specify both fixed effects (e.g., Treatment allocation) and a random effect (e.g., blocking design) across the samples. Genomica therefore carries out a linear mixed model on all the features in the data table and returns a comparison table to summarize the false discovery rate adjusted comparisons throughout. Finally, these significant (*P* adjusted < 0.05; unless otherwise specified by the user) features are selected for functional enrichment analysis, whose outputs are summarized in both enrichment tables and publication-ready figures (e.g., tree plot)

The pipeline requires only two input files (i.e., Data and Metadata), which are used in Genomica to carry out a false discovery rate corrected LMM, thus accounting for multiple hypothesis testing. Users can specify the significance threshold (default *P* adjusted value is 0.05), under which enriched or depleted orthologs in comparison to a control level set by the user, are employed into the FEA (Fig. 1). This is implemented via the R function *genomica*, depicted below (1) where the data frames Data and Metadata, the list of predictors (i.e., fixed effects), the random effect and their levels can be specified. Moreover, users can provide a folder name for the output directory, choose whether to Log_10_ transform Data and set the output image resolution (the default is 300 dpi).

1 $$ \begin{gathered} {\mathrm{genomica}}({\mathrm{Data}}, \;{\mathrm{Metadata}},\;{\mathrm{Predictors}},\;{\mathrm{P}}1\_{\mathrm{Levels}},\;{\mathrm{P}}2\_{\mathrm{Levels}}, \hfill \\ \quad {\mathrm{R}}\_{\mathrm{Effects}},\; {\mathrm{R}}1\_{\mathrm{Levels}},\;{\mathrm{Log}}10\_{\mathrm{Transf}},\;{\mathrm{Folder}}\_{\mathrm{Name}}, \hfill \\ \quad {\mathrm{FDR}}\_{\mathrm{Level}},\; {\mathrm{ImgRes}} ) \hfill \\ \end{gathered} $$

Currently, Genomica allows to use a maximum of two predictors, which can be either numerical (e.g., methane emission) or categorical (e.g., Treatment 1, Treatment 2, Treatment 3), in which case, the users can also specify the predictor levels. This is done via the “P1_Levels” and “P2_Levels” vectors, where the first element of the vector is the control level, thus allowing specific comparisons such as Treatment 2 Vs Treatment 1 and Treatment 3 Vs Treatment 1 of the above example. Moreover, currently Genomica allows one random effect (R_Effects) whose levels (e.g., Block 1, Block 2) are specified by the argument R1_Levels. All the outputs of Genomica are written both as tab delimited.txt and.xlsx files, whilst the figures are saved as.tiff files. The output figures will meet the standard required for publication, thus requiring minimal input from the users during the submission of their work to peer reviewed journals.

If specified, the features in Data are log_10_ (x + 1) transformed before carrying out the LMM via implementing the function *lmer* (2) of the package lme4 [18] to all the KOs with cumulative abundance > 0 (e.g., copies per million).

2 $$ {\mathrm{KO}}\sim{\mathrm{Fixed}}\;{\text{ effect}} 1*{\text{Fixed }}\;{\mathrm{effect}} 2 + (1|{\text{Random }}\;{\mathrm{effect}}) $$

Thereafter, the function *anova* is applied to each *lmer* object, whose output is stored in the “Combined_All_Features” table. The *P* value for the LMM is calculated using the Satterthwaite's method via type III ANOVA through the package lmerTest [19]. In parallel, the false discovery rate (type I error) under repeated testing is calculated with the function *FDR* of the package fuzzySim [20] outputting *P* adjusted values*.* In detail, this is calculated using the Benjamini & Hochberg correction based on the LMM *P* values, leading to a decreased bias due to the large number of variables in the table (e.g., KO features) compared to the number of predictors [21]. At this point, a further LMM is carried out on the significant (*P adjusted* < 0.05; unless otherwise specified by the user) features from the previous step (2), and this time the *summary* function is used to generate a list of comparisons between the different levels of the predictors compared to the control, which is stored in a further table, named the “Significant_Comparisons”. When two predictors are provided, the interaction term between the two predictors (2) are also included in both LMM output tables.

Finally, the KOs significantly (*P adjusted* < 0.05) more or less abundant (i.e., enriched or depleted, respectively) compared to the first element of the “P1_ Levels” and “P2_Levels” vectors are used for the FEA. This is performed via using the function *enrichKO* from the package MicrobiomeProfiler [12], which compares the KO lists generated so far to a background KO data set and therefore grouping KOs by function. The *enrichKO* function is carried out both cumulatively (i.e., all the levels together compared to the control) and level-wise (i.e., one predictor level at the time in comparison to the control), in case of categorical predictors. The functions *pairwise_termsim* and *treeplot* from the package enrichplot [22] are implemented to create a tree plot for the cumulative FEA, when at least 5 functions are found. In parallel, the function *dotplot* of the package clusterProfiler [13, 14] is implemented for the level-wise FEA, outputting a dot plot for the different comparisons.

In parallel, model diagnosis is carried out via implementing the function *shapiro.test(residuals(model))* to check for normality, the function *bptest(residuals(model)* ~ *fitted(model))* from the package lmtest [23] to check for heteroscedasticity and the functions *simulateResiduals()* and *testResiduals()* from the package DHARMa [24] to analyze eventual outliers.

## Results

Genomica is fully implemented in R, and it can be downloaded from https://github.com/sgalg/Genomica. Genomica allows users to discriminate between significantly enriched or depleted functions, starting from the analysis of KOs. Genomica is pre-loaded with “Data_Demo” (DD) and a “Metadata_Demo” (MD) files, which are subset data frames from a parallel metagenomic project (European Nucleotide Archive; accession number PRJEB85873). DD is a data frame of 500 unstratified orthologs across 35 samples, whereas MD provides information on the treatment allocations (i.e., treatments 1 to treatment 5, with treatment 1 being the control) for these 35 samples together with their distribution across the random effect “Block” (i.e., derived from a randomised block design). The data in DD was generated during a metagenomic study assessing the sensitivity of the caecal metagenome of laying hens to an in-feed intervention [25]. The FASTQ reads were processed using Kneaddata [26, 27], enabling low-quality read and adapter removal, as well as host genome decontamination. Microbial functional profiling was then performed using HUMAnN 3.5 [28], generating gene families feature tables that were normalised to copies per million (CPM), and subsequently grouped into KOs within HUMAnN. The original KO dataset comprised 20,257 stratified KOs across 60 samples. However, to facilitate the user experience, a randomly selected subset of 500 unstratified KOs was used to assemble the data frame in DD, which is ultimately made available through Genomica for demonstration purposes.

Since, to the best the author’s knowledge there is no other package that, similarly to Genomica, integrates LMM, FDR and FEA for the study of orthologs, the performance of Genomica and the results in terms of outputs and file organization are described here through the analysis of DD via using *genomica()*:

3 $$\begin{gathered} {\mathrm{genomica}}({\mathrm{Data}}\; = \; {\mathrm{DD}},\;{\mathrm{Metadata}}\; = \;{\mathrm{MD}}, \hfill \\ {\mathrm{Predictors}} \; = \;{\mathrm{c}}\left( {^{\prime}{\mathrm{Treatment}}^{\prime}} \right), \hfill \\ {\mathrm{P}}1_{{{\mathrm{Levels}}}} \; = \;{\mathrm{c}}\left( {^{\prime}1^{\prime},^{\prime}2^{\prime},^{\prime}3^{\prime},^{\prime}4^{\prime},^{\prime}5^{\prime}} \right), \hfill \\ {\mathrm{R}}_{{{\mathrm{Effects}}}} \; = \; {\mathrm{c}}\left( {^{\prime}{\mathrm{Block}}^{\prime}} \right),\; {\mathrm{R}}1_{{{\mathrm{Levels}}}} \; = \;{\mathrm{c}}\left( {^{\prime}1^{\prime},^{\prime}2^{\prime},^{\prime}3^{\prime},^{\prime}4^{\prime},^{\prime}5^{\prime},^{\prime}6^{\prime},^{\prime}7^{\prime}} \right), \hfill \\ {\mathrm{Log}}10\_{\mathrm{Transf}} = {\mathrm{TRUE}}, \hfill \\ {\mathrm{Folder}}\_{\text{Name }} = {\mathrm{c}}\left( {^{\prime}{\mathrm{Results}}^{\prime}} \right),\;{\mathrm{FDR}}\_{\mathrm{Level}} = 0.05,\;{\mathrm{ImgRes}} = 1200) \hfill \\ \end{gathered} $$

As described in detail below, an example of the LMM analysis output and corresponding model diagnostics are provided in Additional files 1–3, while an example of statistical enrichment output is provided in Additional file 4.

The full pipeline for the analysis of the 500 orthologs is completed in 38.73 s on a 13th Gen Intel® Core™ i7-13620H 2.40 GHz, with 32 GB of RAM, whilst the size of the output directory is 3.50 GB, with the currently selected 1200 dpi high-resolution Fig. 3. The “Combined_All_Features” file lists the generic LMM output, which allows to assess the model parameters and performance for each KO (Additional file 1). In this case it can be seen that 7 orthologs (K00185, K00405, K00413, K00459, K00619, K00909, and K01093) were not processed through the LMM, since their abundance was 0 copies per million throughout the samples (pre-*genomica*). Moreover, 180 orthologs were associated to a *P* value lower than 0.05, but only 89 of these were linked to an adjusted *P* value lower than 0.05. Furthermore, the sum of squares, the mean squares, the numerator and denominator degrees of freedom and the F value for the test are also summarized in the table. If two predictors were provided, the table would have included three rows for each observation, two for each predictor and one for their interaction term. The “Significant_Comparisons” table summarizes the output of the function *summary* on the LMM objects generated during the previous step. This table (Additional file 2) shows the significant (*P adjusted* < 0.05) level-wise comparisons to the control group (i.e., treatment 1). Thus, the LMM estimate, standard error, degrees of freedom, t, *P* and *P adjusted* values are summarized in this table, which also includes a description of whether the KOs were found to be enriched (i.e., more abundant) or depleted (i.e., less abundant) in the different comparisons relative to the control. In this case, 16 comparisons across 9 KOs were enriched in comparison to treatment 1, whilst 266 comparisons across 80 KOs were depleted.

In parallel, the folder Model_Diagnosis (Additional file 3), provides the results for the analysis of model residual normality, heteroscedasticity and outliers. This is shown only for the significant comparisons, therefore in this case it can be seen that 23.6% of the analyzed models showed non-normal residuals, whilst 17.98% showed heteroscedasticity and none (0%) showed significant residual outliers. All in all, it can be seen that in none of the models the residuals were non-normal, heteroscedastic and linked to outliers at the same time, in support of the validity of the used approach [29].

**Fig. 2:**
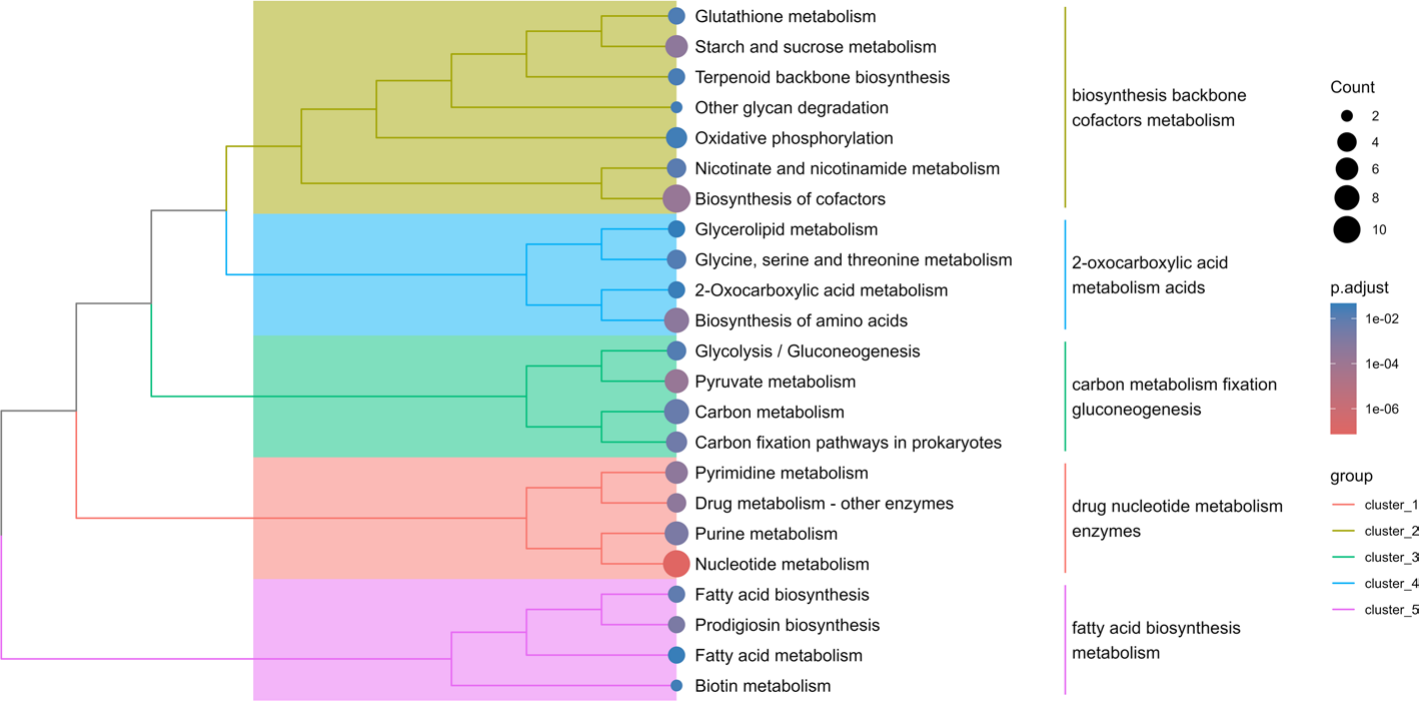
Example of an enrichment tree produced by Genomica, in this case showing the cumulative FEA results of the depleted orthologs starting from the DD data frame. This figure is generated in Genomica via calling the package MicrobiomeProfiler after generating a list of significantly (*P* adjusted < 0.05) differentially abundant orthologs

**Fig. 3:**
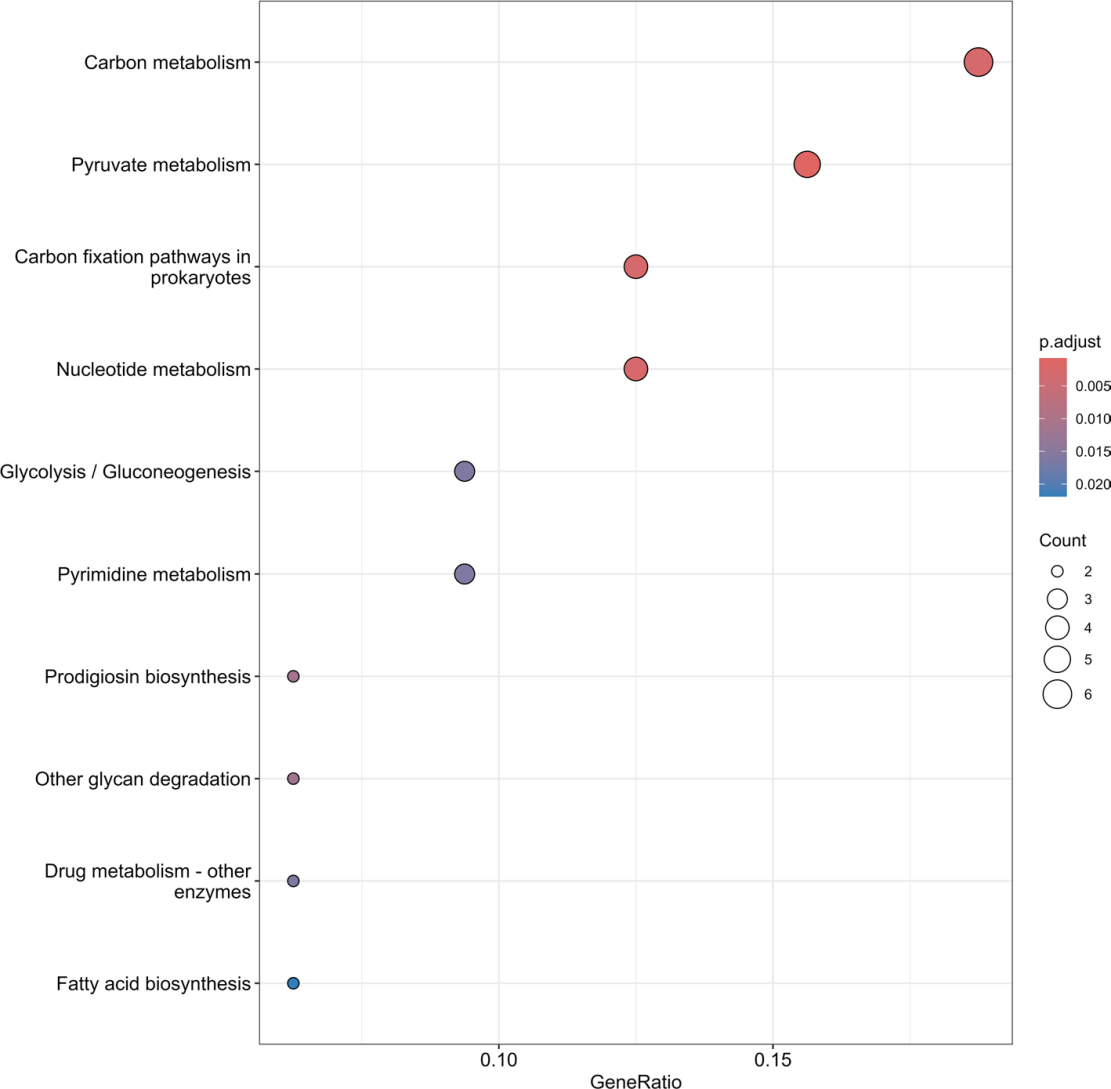
Example of a dot plot produced by Genomica, in this case showing the level-wise FEA of the depleted KOs in Treatment 2 compared to the control (Treatment 1). This is generated in Genomica via calling the package MicrobiomeProfiler

The results of the FEA are summarized in a separated directory, named “Enrichment”, where two sub-directories store the results for the enriched and the depleted KOs. In each of the sub-directories the results of both the cumulative FEA and the level-wise FEA can be found. The cumulative results are stored in the Treatment_Cumulative_Vs_Control tables. To show an example for this output, the Treatment_Cumulative_Vs_Control_Depleted table is depicted in Additional file 4, which provides an overview of the FEA grouped KEGG pathways in the column “Description”, the list of KOs belonging to that pathway (i.e., “Gene ID” column) and the list of the results of the statistical analysis for each specific pathway/function. Finally, the cumulative enrichment tree (Fig. 2) and the dot plot for each level-wise comparison (e.g., Treatment 2 Vs Treatment 1, Fig. 3) are found in the respective directories. Therefore, it can be seen that functions such as fatty acid biosynthesis, gluconeogenesis, or cofactor metabolisms were found to be depleted in all the treatment groups compared to the control. In parallel, amino acids biosynthesis and riboflavin metabolism were more represented in all the treatment groups compared to the control. It is important to note that these observations are specific to the subset analyzed here (i.e., 500 KOs across 35 samples) and are not linked to the findings of the original work from which this subset was generated (European Nucleotide Archive; accession number PRJEB85873) [25].

## Conclusions

Genomica is an R package to analyze orthologs in metagenomic data sets. Its minimal input requirements allow users with any level of expertise in programming to carry out statistical analysis as required for these types of multivariate tests. All in all, Genomica is a tool to assess and enrich differentially abundant functions across treatment layouts, providing an automated protocol for the analysis of biological relevant pathways (e.g., intrinsic and extrinsic antimicrobial resistance) in both surveillance studies and when testing specific interventions.

### Availability and requirements

Project name: Genomica

Project home page: https://github.com/sgalg/Genomica

Operating system(s): Platform independent.Programming language: R

Other requirements: readxl, writexl, lme4, lmerTest, dplyr, fuzzySim, MicrobiomeProfiler, enrichplot, clusterProfiler, tictoc, tidyr, lmtest, DHARMa

License: GPL (>= 3)

Any restrictions to use by non-academics: None.

## Supplementary Information

Below is the link to the electronic supplementary material.

Supplementary Material 1. Additional file 1: Combined_All_Results table, output of the function genomica when analyzing the 500 orthologs from the Data_Demo data frame. This file summarizes the generic LMM output, generated using the function anova in Genomica.

Supplementary Material 2. Additional file 2: Significant_Comparisons table, output of the function genomica when analyzing the 500 orthologs from the Data_Demo data frame. This file summarizes the specific comparisons of each predictor level in comparison to the baseline as specified by the user and is generated in Genomica by using the function summary on each previously generated LMM object.

Supplementary Material 3. Additional file 3: LMM model diagnosis showing the analysis of model residual normality, heteroscedasticity and outliers.

Supplementary Material 4. Additional file 4: “Treatment_Cumulative_Vs_Control_Depleted” table, output of the function genomica, depicting the results of the functional enrichment analysis of the significantly depleted KOs across all the treatment levels in comparison to the control (cumulative functional enrichment analysis).

## Data Availability

The datasets analyzed during the current study are available in the European Nucleotide Archive (ENA) repository, linked to the accession number PRJEB85873. In parallel, the demo data sets used in this study are pre-loaded in the R package Genomica.
